# The complete mitochondrial genome of the *Goniopora lobata*

**DOI:** 10.1080/23802359.2020.1715285

**Published:** 2020-01-27

**Authors:** Huipai Peng, Yanping Zhang, Cheng Shen, Li Liu

**Affiliations:** a Fisheries College, Guangdong Ocean University, Zhanjiang, China;; b XuWen National Coral Reef Nature Reserve, Zhanjiang, China;; c Southern Marine Science and Engineering Guangdong Laboratory, Zhanjiang, China

**Keywords:** *Goniopora lobata*, coral, mitogenome

## Abstract

In this study, the complete mitogenome sequence of the *Goniopora lobata* has been sequenced using next-generation sequence method. The overall of *G. lobata* mitogenome is 25.72% for A, 13.59% for C, 23.42% for G, and 37.27% for T, as well as 37.01% for low GC. The assembled mitogenome, consisting of 18770 bp, has 13 unique protein-coding genes (PCGs), three transfer RNA genes, and two ribosomal RNA genes. The complete mitogenome of *G. lobata* provides essential and important DNA molecular data for further phylogenetic and evolutionary analysis of stony coral phylogeny.

*Goniopora lobata* is a part of the family of Goniopora, which is widely distributed throughout the Indo-Pacific and South China Sea. They usually forms large single-species stands, especially in turbid water. The colonies of these species are hemispherical or, more usually, form short thick columns. Columellae and oral cones are small. Polyps are elongate when fully extended. The color of these species is usually brown, yellow or green, often with contrasting oral cones, and tentacle tips (Veron and Stafford-Smith 2000). The first establishment of *G. lobata* mitogenome is meaningful for further evolutionary and phylogenetic analysis of stony coral.

Samples (stored in Aquatic Organisms Museum of Guangdong Ocean University with voucher no. R28-8) of *G. lobata* were collected from XuWen sea area (109°50′12″–109°56′24″E, 20°10′36″–20°27′00″N) in Zhanjiang, China. We used next-generation sequencing to perform whole genome sequencing with assembly guided by reference sequence (NC_015643.1 *Goniopora columna* mitochondrion).

The complete mitogenome of G. lobata was 187,70 bp in size and its overall base composition is 25.72% for A, 13.59% for C, 23.42% for G, and 37.27% for T, and with 37.01% content low GC. Circular form of the complete mitogenome was about 776× coverage on average.

The protein-coding, rRNA, and tRNA genes of *G. lobata* were annotated using MITOS (Bernt et al. 2013) and manually inspected. The complete mitogenome of *G. lobata* includes 13 unique protein-coding genes (PCGs), three transfer RNA genes, and two ribosomal RNA genes. All genes including 13 PCGs share the start codon ATG, except for COX2, COX3, NAD5 (with GTG start codon), and ATP6 (with AAG start codon). All the 13 PCGs share the stop codon TAA, except for NAD2, NAD4, COX2 (with TAG stop codon), NAD4L (with GAA stop codon), and ATP6 (with AAA stop codon). Among 13 PCGs, the longest one is NAD5 gene (13013 bp), whereas the shortest is ATP8 gene (216 bp). The size of small ribosomal RNA (12S rRNA) and large ribosomal RNA (16S rRNA) gene are 1117 bp and 2008 bp, respectively.

To validate the phylogenetic position of *G. lobata*, we used MEGA 7.0 software (Kumar et al. 2016) to construct a maximum-likelihood tree (with 1000 bootstrap replicates and GTR + G model) containing complete mitogenomes of 18 species derived from 13 different families in Scleractinia. *Corallimorphus profundus* derived from Corallimorphidae was used as outgroup related to *G. lobata* coral with high bootstrap value supported (Figure 1). In conclusion, the complete mitogenome of *G. lobata* deduced in this study provides essential and important DNA molecular data for further phylogenetic and evolutionary analysis for stony coral phylogeny.

**Figure 1.**
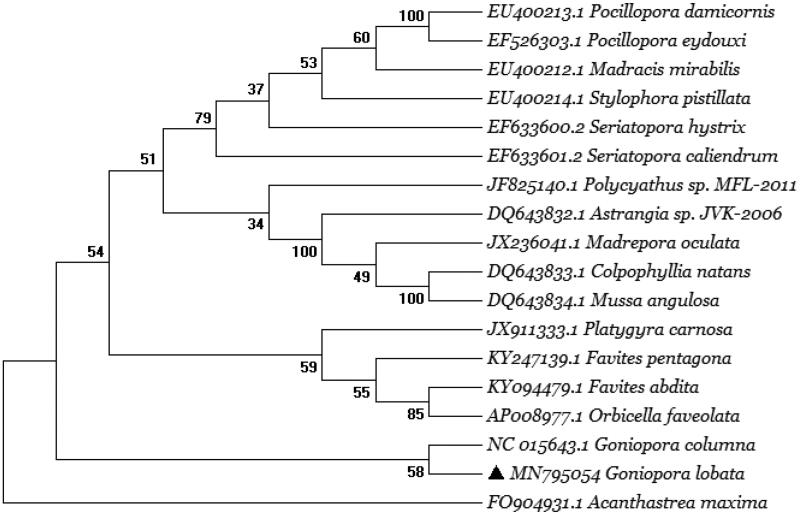
Molecular phylogeny of *Goniopora lobata* and other related species in Scleractina based on complete mitogenome. The complete mitogenome is downloaded from GenBank, and phylogenic tree is constructed using the maximum-likelihood method with 1000 bootstrap. The gene’s accession number for tree construction is listed as follow: *Pocillopora eydouxi* (EF526303.1), *Pocillopora damicornis* (EU400213.1), *Madracis mirabilis* (EU400212.1), *Stylophora pistillata* (EU400214.1), *Seriatopora caliendrum* (EF633601.2), *Seriatopora hystrix* (EF633600.2), *Polycyathus sp. MFL-2011* (JF825140.1), *Astrangia sp.JVK-2006* (DQ643832.1), *Madrepora oculata* (JX236041.1), *Colpophyllia natans* (DQ643833.1), *Mussa angulosa* (DQ643834.1), *Goniopora columna* (NC0156434.1), *Goniopora lobata* (MN795054), *Platygyra carnosa* (JX911333.1), *Favites pentagon* (KY247139.1), *Favites abdita* (KY094479.1), *Orbicella faveolata* (AP008977.1), *Acanthastrea maxima* (FO904931.1).
